# Bioactive metabolites of *Blumea lacera* attenuate anxiety and depression in rodents and computer‐aided model

**DOI:** 10.1002/fsn3.2362

**Published:** 2021-05-31

**Authors:** Md. Amjad Hossen, A.S.M. Ali Reza, Md. Badrul Amin, Mst. Samima Nasrin, Tawhidul Amin Khan, Md. Habibur Rahman Rajib, Abu Montakim Tareq, Md. Anwarul Haque, Md. Atiar Rahman, Md. Areeful Haque

**Affiliations:** ^1^ Department of Pharmacy International Islamic University Chittagong Chittagong Bangladesh; ^2^ Department of Biochemistry and Molecular Biology Faculty of Biological Sciences University of Chittagong Chittagong Bangladesh; ^3^ Department of Pharmacy, Faculty of Science University of Rajshahi Rajshahi Bangladesh; ^4^ Department of Experimental Pathology, Faculty of Medicine University of Tsukuba Ibaraki Japan; ^5^ Faculty of Pharmacy Universiti Kebangsaan Malaysia Kuala Lumpur Malaysia

**Keywords:** antidepressant, antioxidant effects, anxiolytic, *Blumea lacera*, gamma‐sitosterol, thymol

## Abstract

*Blumea lacera* is an edible plant with imperative medicinal values. However, the anxiolytic and antidepressant roles of *B. lacera* have not been well‐explained. Therefore, the current study aims to explore the impending bioactive metabolites and roles of *B. lacera* methanol leaf extract (Me‐BLL) in attenuating anxiety and depression through several experimental and computer‐aided approaches. The chemical characterization of Me‐BLL was performed through standard phytochemical and GC‐MS analyses. To explore the neuropharmacological insights, Swiss albino mice were treated with Me‐BLL at doses of 200–400 mg/kg, p.o. The anxiolytic effects were observed employing elevated plus maze (EPM), light–dark box (LDB), and hole‐board (HBT) tests, while antidepressant effects were evaluated using forced swimming (FST) and tail suspension tests (TST). Diazepam (1 mg/kg, i.p.) and fluoxetine HCl (20 mg/kg, p.o.) were used as the reference standard. The phytochemical analyses revealed several bioactive metabolites, including higher contents of total phenolics and flavonoids. The EPM and LDB tests demonstrated an increased time spent in open arms and light box, and the HBT showed an increased number of head dipping, indicating the anxiolytic effects of Me‐BLL. The TST and FST revealed a decrease in immobility time, meaning the persuasive antidepressant effects. The antioxidative effects of Me‐BLL have also been observed prominently. Correspondingly, the computer‐aided investigation confirmed several bioactive lead molecules. Specifically, thymol and cuminol revealed potential anxiolytic and antioxidant effects, while stigmast‐5‐en‐3.beta.‐ol and gamma‐sitosterol possessed promising antidepressant effects. Taken these results as a base, the plant has imperative potentials in managing anxiety and depression‐like disorders.

## Graphical abstract

Chemical analysis revealed wide range of bioactive metabolites including phenolics and flavonoids in Me‐BLL. Me‐BLL produced promising anxiolytic effects in EPM, LDB, and HBT in experimental rodents. Me‐BLL revealed significant reduction of immobility time in FST and TST. Lead molecules like cuminol, thymol, and γ‐sitosterol revealed potentials in computer‐aided model.

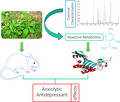

## 1 INTRODUCTION

More than 450 million people suffer from psychiatric or behavioral disorders, which add up to 12.3 percent of the global disease burden and are expected to increase to 15 percent by 2020 (Rajput et al., 2019). Currently, the most psychiatric conditions that raise morbidity in the world tend to be anxiety and depression. In addition, the WHO has rated depressive disorders as the world's leading source of non‐fatal health conditions, with anxiety disorders ranking sixth (WHO, 2017). Multiple studies indicated that both anxiety and depression happen simultaneously and do not represent distinct disease entities. Around one portion of those researched with depression are also diagnosed with anxiety disarray.

The leading causes of anxiety and depression remain a great conundrum. Still, few prevalence factors like genetic, environmental, biological, and psychological have been unfolded to be connected in the progression of such neuropsychiatric disorders (Berton & Nestler, 2006). Various pathophysiological events occur as a result of changes in the γaminobutyric acid (GABA)ergic, serotoninergic, and glutamatergic systems. The GABA acts as the vital modulator mechanism in the central nervous system (CNS), an impact that is balanced by glutamate (with an excitatory activity). A down‐regulation of GABAergic transmission has been linked to anxiety disorders; thus, many anxiolytic drugs have this system as a target of action. Its activity results from the recognition of two types of receptors: the ionotropic GABA‐A and metabotropic GABA‐B receptors. Many physicians prescribed benzodiazepines as anxiolytics that act on the GABA‐A, but these generate many adverse effects such as sedation, motor incoordination, cognitive impairments, tolerance, and addiction (Barker et al., 2004). Besides, the 5‐hydroxytryptamine (5‐HT) is a key modulatory neurotransmitter associated with the pathophysiology and treatment of anxiety (Resstel et al., 2009). Therefore, the antidepressants which modulate the 5‐HT reuptake (SSRIs) are acted as anxiolytics. Notably, the 5‐HT1A receptor is widely expressed, and it is associated with anxiety disorders.

However, present top‐notch neuropsychiatric medications (e.g., benzodiazepines, selective serotonin, and/or serotonin‐norepinephrine reuptake inhibitors) cannot offer adequate clinical procedures to simultaneously modulate anxiety, depression, chronic inflammation, and reactive oxygen species (ROS) (Penn & Tracy, 2012). In addition, these medications have adverse side effects, including sedation, sexual dysfunction, memory disruptions, amnesia, and daytime drowsiness, which was of considerable concern to society (Wang et al., 2018). Throughout the quest for new remedial products for the treatment of neurological disorders, herbal/medicinal plant research has also led mainly to demonstrating the pharmacological efficacy of various herbs throughout different animal models (Zhang, 2004). In addition, the discovery of potentially bioactive compounds from herbal products has broad targets of pharmacological responses that are the focal point of recent global research interest (Lee & Kim, 2016). As developments of the drug are involved in the discovery of lead compounds from natural products, while it was followed by lead identification, lead optimization, lead development, finally it approaches for successfully consecutive clinical trials, and the compounds also approved for clinical application (Balunas & Kinghorn, 2005).

*Blumea lacera* (Burm.f.) DC., belonging to the Asteraceae family, has an enormous medicinal value, and the leaves have been widely used in the traditional medicinal system. The leaf is edible and the most used part of the herb. The juice of leaves is antispasmodic, anthelmintic, astringent, febrifuge, stimulant, and diuretic; and able to cure bronchitis, fevers, and burning sensation (Khare, 2008). Nonetheless, so far, little work has been conducted to explore its pharmacological potential. Therefore, this research examined the antioxidant, anxiolytic, and antidepressant activity of *B. lacera* systematically alongside its chemical characterization and pursued computer‐aided approaches to unravel the possible bioactive lead molecules of the plant.

## 2 MATERIALS AND METHODS

### 2.1 Chemicals

Methanol, *n*‐Hexane, petroleum ether, ethyl acetate, Bouin's fluid, formalin, DPPH, O‐phenanthroline, potassium ferricyanide, tri‐chloroacetic acid, EDTA, and sodium dihydrogen phosphate were procured from Sigma‐Aldrich (St. Louis, USA). Diazepam and Fluoxetine hydrochloride were purchased from Square Pharmaceutical Ltd. (Dhaka, Bangladesh). Tween‐80 was obtained from Scharlab (Sentmenat, Barcelona, Spain). Aluminum chloride, Folin–Ciocalteu reagent, potassium acetate, sodium carbonate, gallic acid, isopropyl alcohol, ferric chloride, ferrous sulfate, sodium salicylate, hydrogen peroxide, and quercetin were bought from Merck (Darmstadt, Germany).

### 2.2 *Plant* *materials*

Fresh leaves of *B. lacera* were collected from the Bayazid Bostami, Chittagong, Bangladesh. The plant sample was identified by a taxonomist Prof. Dr. Shaikh Bakhtiar Uddin, Department of Botany, University of Chittagong, Bangladesh. A voucher specimen (BOT‐T1017) was deposited in the Herbarium center of the University of Chittagong, Bangladesh.

### 2.3 Animals

Five to six weeks old male Swiss albino mice weighing between 22 to 30 g were acquired from the animal research division of the International Centre for Diarrheal Disease and Research, Bangladesh (ICDDR,B). Animals were housed in poly‐carbonated cages, ensuring a standard laboratory condition (room temperature 23 ± 2℃) and humidity 55%–60% in a 12 hr/daylight cycle (Akter et al., 2021). They had free access to diet and tap water supplied with pellets. The mice had been acclimatized (14 days) to adapt to the laboratory environment before starting the experiments.

### 2.4 *Extract* *preparation*

The collected leaves were cleaned and dried under shade ground at room temperature (23 ± 0.5℃). The dried samples ground to a coarse powder with a mechanical grinder, and then, around 500 g powder successively extracted with methanol to yield crude extract. Finally, the filtrate was evaporated by a rotary evaporator (RE200, Bibby Sterling, UK) under reduced pressure and temperature below 50℃. The methanol extract (Me‐BLL) yield value was noted as 16 g, and until the experiment was conducted, Me‐BLL was stored at 4℃ (Hossen et al., 2021).

### 2.5 *Qualitative* *phytochemical* *screening*

The qualitative phytochemical analysis has been performed following the standard protocol (Akter et al., 2021; Khan et al., 2020) to confirm the presence of various secondary metabolites in Me‐BLL.

### 2.6 *Quantitative* *phytochemical* *analysis*

#### 2.6.1 GC‐MS analysis

The bioactive compounds of Me‐BLL extract were investigated through GC‐MS with electron impact ionization (EI) method on gas chromatography (GC‐17A, Shimadzu Corporation, Kyoto, Japan), coupled with a mass spectrometer (GC‐MS TQ 8040, Shimadzu Corporation, Kyoto, Japan). The fused capillary silica column (Rxi‐5 ms; 0.25 m film, 30 m long, and 0.32 mm internal diameter) was coated with DB‐1 (J&W). The temperature of the inlet was 260℃ and the oven set at 70℃ (0 min); 10℃, 150℃ (5 min); 12℃, 200℃ (15 min); 12℃, 220℃ (5 min) with a holding period of 10 min. The flow rate of the column was 0.6 ml/min Helium gas at a constant pressure of 90 kPa. The aux (GC to MS interface) temperature was 280℃. The MS was set with a scanning mode with a scanning range of 40–350 amu, while the ionizing mode was in the form of electron ionization (EI) and the range of mass set within 50–550 m/z. One μL of the sample was injected in split fewer modes. Complete GC‐MS running time was set for 29.33 min, and the compounds in the peak areas were identified by comparison to those in the GC‐MS library version NIST 08‐S database.

#### 2.6.2 Determination of total plant phenolics

The concentrations of phenolic compounds in Me‐BLL were determined following the established method (Ali Reza et al., 2018; Esmaeilzadeh Kenari et al., 2014). The 0.5 ml of plant extract or standard solution at different concentrations was added to 2.5 ml of Folin–Ciocalteu (diluted ten times with water) reagent and 2.5 ml of Na_2_CO_3_ (7.5%) solution. The reaction mixture was incubated for 20 min at 25℃, and the absorbance of the mixture was measured at 760 nm. The experiment was performed in triplicate, and findings were stated as mean ± *SEM*, and values are expressed as mg of gallic acid equivalent (GAE)/g of dried extract.

#### 2.6.3 Determination of total plant flavonoids

The total flavonoid contents of the Me‐BLL were investigated by the aluminum chloride colorimetric method described previously (Ali Reza et al., 2018; Rajaei et al., 2021). One mL from each concentration of the plant extract was added to 3.0 ml of methanol, 0.2 ml of 10% AlCl_3_, 0.2 ml of 1 M potassium acetate, and 5.6 ml of distilled water. The reaction mixture was then incubated at room temperature for 30 min to complete the reaction. The absorbance of the mixture was measured at 420 nm. The study was conducted in triplicate, and results were reported as mean ± *SEM*, and values are expressed as mg of quercetin equivalent per gram (QE/g) of dried extract.

### 2.7 *Evaluation* *of antioxidative* *potential*

#### 2.7.1 DPPH free radical scavenging assay

The free radical scavenging activity of Me‐BLL was determined by DPPH assay according to the method described previously (Li et al., 2019; Rashid Chowdhury et al., 2021). Two mL of methanol solution of plant extract or reference standard ascorbic acid at different concentrations was mixed with 3 ml of methanol solution of DPPH (4 mg in 100 ml methanol) into the test tube. The reaction mixture was incubated at room temperature for 30 min in a dark place to complete the reaction. The absorbance of the solution was measured spectrophotometrically at 517 nm. DPPH free radical scavenging ability (%) was calculated by using the formula:

$$\left[\left(\text{absorbance of control}‐\text{absorbance of sample}\right)/\text{absorbance of control}\right]\times 100.$$

#### 2.7.2 Hydroxyl radical scavenging assay

The hydroxyl radical scavenging action of Me‐BLL was determined by the method as described earlier (Ali Reza et al., 2018; Haida & Hakiman, 2019). A 3 ml of the reaction mixture was prepared with 1 ml of 1.5 mM FeSO_4_, 0.7 ml of 6 mM H_2_O_2_, and 0.3 ml of 20 mM sodium salicylate to dilute at 50–800 μg/mL. The mixture was read at 562 nm after one h incubation at 37℃ against an appropriate blank solution. Hydroxyl radical scavenging ability (%) was calculated by using the formula:

$$\left[\left(\text{absorbance of control}‐\text{absorbance of sample}\right)/\text{absorbance of control}\right]\times 100$$

#### 2.7.3 Assay of iron‐chelating effect

The iron‐chelating effect of Me‐BLL was evaluated compared with ascorbic acid, and also the whole test was administrated according to the established procedure (Akhter et al., 2015). Briefly, both the test sample and ascorbic acid (50–800 μg/mL) were added to O‐phenanthroline (0.05%) and ferric chloride (200 μM) solution while the crude samples excluded in control. The reaction mixture was read at 510 nm after 10 min incubation at room temperature. The following equation calculated the percentage of iron‐chelating activity of Me‐BLL:

%of chelating activity=[(Test absorbance‐control)/Test absorbance]×100.

### 2.8 *Acute* *oral toxicity* *test*

The acute oral toxicity test was performed following the OECD guidelines and established protocol. The allocated animals (*n* = 5) were administered a single oral dose (100 to 2000 mg/kg, body weight) of the test extract (Me‐BLL). Prior to administering the extract, mice were kept fasting overnight, and food was also delayed between 3 and 4 hr. After administration, food was withheld for a further 3–4 hr. Experimental animals were observed individually during the first 30 min after dosing, periodically for the first 24 min (special attention for the first 4 hr), with special monitoring for possible unusual responses including behavioral changes, allergic syndromes (itching, swelling, skin, and rash), and mortality over the next 72 hr. The median therapeutic effective dose was intervened as one‐tenth of the median lethal dose (LD50 >2.0 g/kg) (Al‐Araby et al., 2020).

### 2.9 *Experimental* *design*

For each experiment, a total of twenty mice were separated into four groups (Group‐I to IV) containing five mice (*n* = 5) in each group. The control group (Group‐I) received vehicle (1% Tween‐80 in water, 10 ml/kg, p.o.), whereas the reference drug group (Group‐II) received diazepam (1 mg/kg, b.w., i.p.) for EPM, HBT, and LDB tests and fluoxetine HCl (20 mg/kg, b.w., p.o.) for TST and FST, respectively. In addition, the treatment groups (Group‐III & IV) were administrated with Me‐BLL at doses of 200 and 400 mg/kg, b.w., p.o., respectively. All the experiments were observational, so no dissection/surgery was required or any experimental animals needed to sacrifice.

### 2.10 *Anxiolytic* *activity*

#### 2.10.1 Elevated plus maze test

The elevated plus maze test (EPM) apparatus was constructed with two open arms (35 × 5 cm^2^), two closed arms (35 × 20 cm^2^), and a central square (plus sign) (5 × 5 cm^2^). The apparatus was positioned at a height of 25 cm above the floor with an open ceiling. The randomly distributed animals were administrated with samples as per the mentioned experimental design. After thirty minutes, each mouse was placed at the central position with the head facing one of the closed arms and observed for 6 min while the last 5 min were counted for anxiolytic behavior followed by adjustment for the first min. During the investigation, open arms entrance and total time spent were recorded (Barua et al., 2012; Goni et al., 2020). Percentage (%) of entries in the open arm = [(Number of entries in the open arm)/(Number of entries in the open arm +Number of entries in the closed arm)] × 100.

#### 2.10.2 Light and dark box test

The light and dark box test (LDB) test helps in predicting whether the anxiolytic or anxiogenic properties are present in laboratory animals. The test was performed following the established method described earlier (Islam et al., 2021). An animal activity monitor outfitted with two‐compartment test chambers housed in a dark, air‐conditioned room was used to conduct the test. The light–dark box is designed with an open‐topped rectangular box consisting of a floor area of 46 × 27 ×30 cm^3^ that is divided into two parts, small (19 × 27 cm^2^) and a large (27 × 27 cm^2^) area. An 8 × 8 cm passageway provided access between the two compartments. Each light compartment was illuminated with a 60W red tungsten bulb at the height of 30 cm above the test chamber's door. The time spent in the compartments was monitored and recorded. Animals (*n* = 5) of each group were administrated as per the experimental design. After 30 min treatment with control, diazepam, and treatment group, each mouse was set onto individually in the test chambers, and their activity was recorded over 5 min.

#### 2.10.3 Hole‐board test

The method applied for hole‐board test (HBT) was similar to those described earlier (Islam et al., 2021; Rashid Chowdhury et al., 2021). A grid pattern with sixteen holes (diameter 3 cm) in this model included a flat platform with an enclosed area (20 x 40 cm^2^) used as an experimental apparatus set up 15 cm above the floor. Dosing treatments have been followed for each group of animals according to the experimental design indicated. The experimental animals were placed in the center of the board thirty minutes after the test dose was administered and permitted free movement. Finally, the head dipping through the holes and the latency of mice dipping the head was counted for 5 min.

### 2.11 *Antidepressant* *activity*

#### 2.11.1 Tail suspension test

Tail suspension test (TST) is a commonly used behavioral model to evaluate the antidepressant activity in mice. Following administration of all the doses as described in the experimental design section, mice were induced in a state of depression (immobility), hanging with adhesive tape at the end of their tail (about 1 cm from the tip of the tail). The total immobility time for each mouse of all groups was recorded during the last 4 min of 6 min (Khan et al., 2020).

#### 2.11.2 Forced swimming test (FST)

According to this method, mice were forced to swim independently in an open glass compartment (10 × 15 cm^2^, d × h) containing fresh water with a depth of 19 cm and kept at (25 ± 1) °C. Mice of all groups were treated as per the statement of the experimental design section. After thirty minutes, each mouse was placed in the tank for 6 min, where the first 2 min was considered initial adjustment time, and the next 4 min was recorded as the immobility duration (Islam et al., 2021).

### 2.12 *Computational* *studies*

#### 2.12.1 Molecular docking analysis

##### Docking tools

The molecular docking was performed using Schrodinger suites‐Maestro 2017‐1. The Pockdrug online server was used to predict the best binding pocket and probable drug ability. Discovery studio (v 4.1) was used for the visualization.

##### Ligand preparation

The chemical structures of eight major compounds were extracted from the PubChem repository (https:/pubchem.ncbi.nlm.nih.gov/). The ligand was prepared using the LigPrep tool, embedded in Schrödinger suite‐Maestro v 11.1, where the following parameters were used for minimization: neutralized at pH 7.0 ± 2.0 using Epik 2.2 and the force field OPLS3.

##### Receptor/Enzyme preparation

Three‐dimensional crystallographic structures of enzyme/receptors were obtained from the Protein Data Bank RCSB PDB: urate oxidase (Uox) enzyme receptor (PDB: 1R4U), potassium channel receptor (PDB: 4UUJ), and human serotonin receptor (PDB: 5I6X). Preprocessing, optimization, and minimization processes were done by using Protein Preparation Wizard. This process is included in the Schrodinger suit‐maestro (v11.1). The structures were optimized at pH 7.0, and water molecules fewer than 3 H‐bonds to non‐waters were removed. Restrained minimization was done where heavy atoms are converged to an RMSD of 0.30 Å on the implemented OPLS3 force field. Then the receptor grids were generated after selecting the best binding sites by using an online tool, PockDrug (Hussein et al., 2015).

##### Glide ligand molecular docking

The molecular docking was performed to select the better ligand that can further be studied comparing with the standard drugs for determining the antioxidant, anxiolytic, and antidepressant effects. The docking was done by ligand docking option using Schrodinger suite‐maestro (v11.1) (Islam et al., 2021).

#### 2.12.2 Evaluation of pharmacokinetic parameters

The absorption, distribution, metabolism, excretion, and toxicity (ADME/T) properties analysis of Me‐BLL were evaluated by the Lipinski's rule of fives and Veber's rules (number of rotatable bonds; topological polar surface area). In addition, the ADME/T properties analysis was evaluated by SwissADME (http://www.swissadme.ch/) (Sakib et al., 2021).

#### 2.12.3 Determination of toxicological properties

AdmetSAR online tool was used to determine the toxicological properties of the selected compounds, while a prime concern during the development of new drugs is toxicity (Yang et al., 2019). In this study, ames toxicity, carcinogenic properties, acute oral toxicity, and rat acute toxicity were predicted.

### 2.13 *Statistical* *analysis*

The data were analyzed by one‐way analysis of variance (ANOVA) followed by Dunnett's test using the GraphPad Prism Version 8.0 (GraphPad Software Inc, San Diego, CA). All the values were expressed as mean ±standard error of the mean (*SEM*). *p* values *<0*.*05* and *<0*.*01* were considered statistically significant.

## 3 RESULTS

### 3.1 *Qualitative* *phytochemical* *screening*

The qualitative phytochemical screening of Me‐BLL unveiled the presence of several secondary metabolites, including alkaloids, carbohydrates, flavonoids, phenols, protein, and amino acids, diterpenes in Me‐BLL (Table 1).

**TABLE 1:** Preliminary phytochemical screening of Me‐BLL

Sl.	Phytochemicals	Name of the tests	Observation
1.	Alkaloids	Mayer's test	++
2.	Carbohydrates	Molisch's test	+
3.	Glycosides	Modified Borntrager's Test	+
4.	Flavonoids	Alkali test	+
5.	Tannins	Gelatin Test	−
6.	Saponins	Froth Test	−
7.	Phenols	Ferric Chloride Test	+
8.	Proteins and amino acids	Xanthoproteic Test	++
9.	Diterpenes	Copper acetate Test	++
10.	Phytosterols	Salkowski's Test	++

Note

Signs indication: (+) = Present, (++) = Abundantly present, (‐) = Absent.

### 3.2 *GC‐MS* *analysis*

**FIGURE 1:**
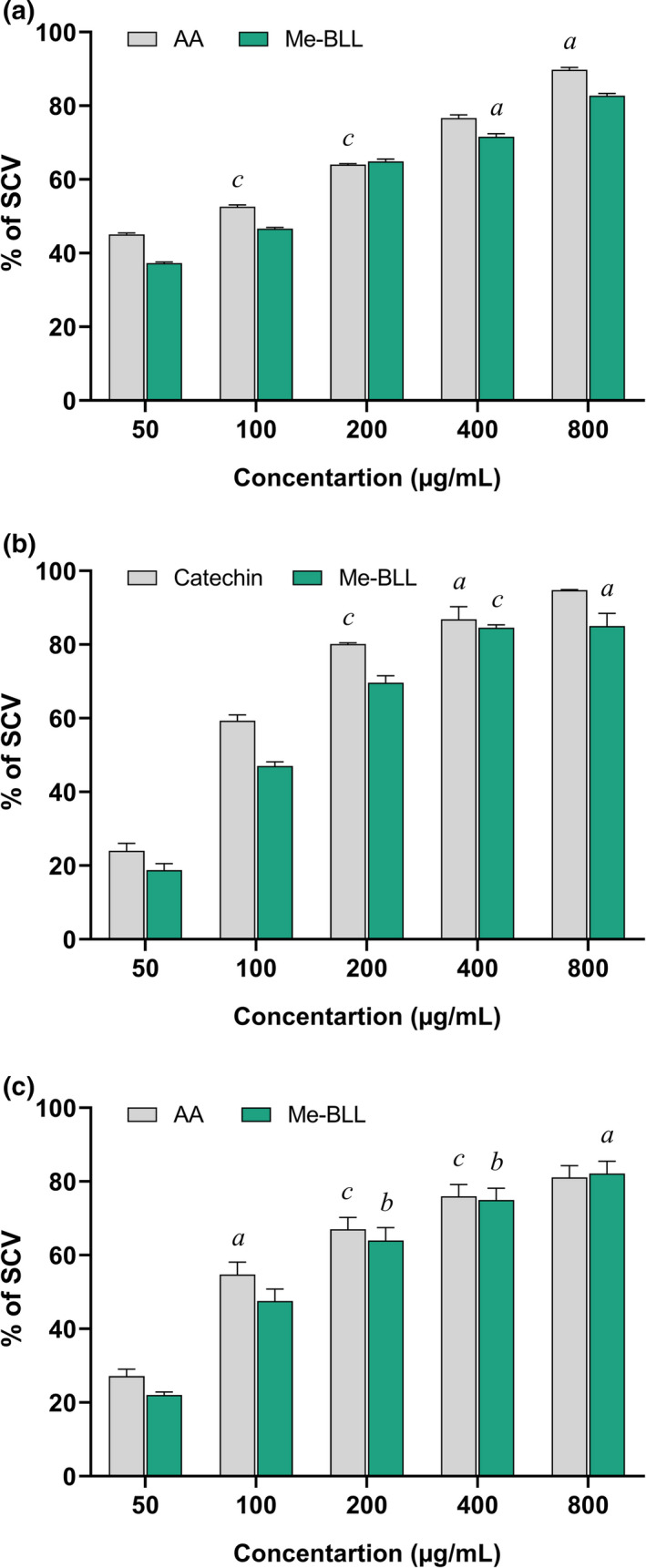
(a) DPPH, (b) HRSA, and (c) ICA activities of Me‐BLL and reference standard in different concentrations. All the values are expressed as mean ± *SEM* (*n* = 3). AA, ascorbic acid; Me‐BLL, methanol extract of *Blumea lacera*; DPPH, 2,2‐diphenyl‐1‐picrylhydrazyl; HRSA, hydroxy radical scavenging activity; ICA, iron‐chelating activity

The GC‐MS analysis of Me‐BLL revealed around thirty secondary metabolites having retention time between 9.16 and 29.09 as listed in Table 2, while the chromatogram is shown in Figure 1. The major secondary metabolites identified in the Me‐BLL include cholest‐5‐en‐3‐ol (3.beta.) (8.29%), beta‐iraldeine (8.20%), stigmasterol (4.40%), 13‐Docosenamide, (Z)‐(1.63%), Gamma‐Sitosterol (0.71%), Squalene (0.56%), Vitamin E (0.41%), Stigmast‐5‐en‐3.beta.‐ol (0.31%), Cuminol (0.23), and Thymol (0.10%).

**TABLE 2:** Secondary metabolites identified in Me‐BLL by GC–MS

Sl.	RT (min)	Compounds	MF	MW (g/mol)	Area (%)	Chemical class
1.	9.16	Cuminol	C10H14O	150.22	0.23	Phenol
2.	10.96	beta‐Caryophyllene	C15H24	204.35	0.43	Bicyclic sesquiterpene
3.	12.65	Thymol	C10H14O	150.22	0.10	Monoterpenoid phenol
4.	13.82	beta‐Turmerone	C15H22O	218.33	1.28	Sesquiterpenoid
5.	16.63	Neophytadiene	C20H38	278.5	0.29	Diterpene
6.	16.93	(‐)‐Isolongifolol	C15H26O	222.37	0.07	Sesquiterpene alcohol
7.	17.25	Hexadecanoic acid, methyl ester	C17H34O2	270.5	2.19	Fatty acid
8.	19.25	Linoleic acid, methyl ester	C19H34O2	294.5	4.67	Fatty acid methyl ester
9.	19.33	9‐Octadecenoic acid, methyl ester, (E)‐	C19H36O2	296.5	6.29	Fatty acid
10.	19.42	Phytol	C20H40O	296.5	1.28	Acyclic diterpene alcohol
11.	20.60	Stigmast−5‐en−3.beta.‐ol	C29H50O	414.7	0.31	Phytosterol
12.	20.66	Gamma‐Sitosterol	C29H50O	414.7	0.71	Phytosterol
13.	21.70	9‐Octadecenamide, (Z)‐	C18H35NO	281.5	0.20	Amines
14.	21.83	2‐Methyl‐Z,Z−3,13‐octadecadienol	C19H36O	280.5	0.15	Enol
15.	22.13	9,12‐Octadecadienoic acid (Z,Z)‐	C18H32O2	280.4	0.28	Fatty acid
16.	22.28	Octadec−9‐enoic acid	C18H34O2	282.5	0.14	Fatty acid
17.	22.43	.beta.‐Iraldeine	C14H22O	206.32	8.20	Unsaturated alicyclic ketone
18.	22.94	Methyl 20‐methyl‐heneicosanoate	C23H46O2	354.6	0.33	Fatty acid
19.	23.08	Widdrol	C15H26O	222.37	3.34	Sesquiterpene
20.	23.18	Capsaicin	C18H27NO3	305.4	0.95	Capsaicinoids
21.	23.36	Dihydrocapsaicin	C18H29NO3	307.4	0.74	Capsaicinoids
22.	23.86	Olein, 1‐mono‐	C21H40O4	356.5	0.30	Lipid
23.	24.07	6‐Octadecenoic acid, (Z)‐	C18H34O2	282.5	0.95	Monosaturated fatty acid
24.	24.28	Methyl tetracosanoate	C25H50O2	382.7	0.65	Fatty acid methyl ester
25.	24.63	13‐Docosenamide, (Z)‐	C22H43NO	337.6	1.63	Amines
26.	24.85	Squalene	C30H50	410.7	0.56	Polyunsaturated hydrocarbon
27.	25.34	Cholest−5‐en−3‐ol (3.beta.)‐	C27H46O	386.7	8.29	Organic compound
28.	25.37	3‐Methyl−2‐butenoic acid	C14H22O2	222.32	7.22	Unsaturated carboxylic acid
29.	27.52	Vitamin E	C29H50O2	430.7	0.41	Organic compound
30.	29.09	Stigmasterol	C29H48O	412.7	4.40	Unsaturated phytosterol

Abbreviations: MF, molecular formula; MW, molecular weight; RT, retention time.

### 3.3 *Total* *plant phenolics and* *flavonoids*

Quantitative analyses revealed that the Me‐BLL contained the highest phenolic content (32.27 ± 1.06 mg of GAE/g of dried extract). At the same time, Me‐BLL also has the highest flavonoid content (172.35 ± 0.25 mg of QE/g of dried extract) (Table 3).

**TABLE 3:** Quantitative analysis of antioxidant‐relevant phytochemicals of Me‐BLL

Antioxidative indices	Me‐BLL
Total phenolic content (mg QE/g crude extract)	32.27 ± 1.067
Total flavonoid content (mg GAE/g crude extract)	172.35 ± 0.24

Abbreviations: GAE, gallic acid equivalent; Me‐BLL, methanol extract of *B. lacera* leaves; QE, quercetin equivalent.

### 3.4 *Antioxidant* *activity*

#### 3.4.1 DPPH free radical scavenging assay

The study explored significant scavenged DPPH radicals in a dose‐dependent manner (Figure 1a). Me‐BLL showed the most potential scavenging activity against DPPH radicals with an IC_50_ value of 133.48 ± 3.67 μg/mL, which is comparable to that of the reference standard ascorbic acid (AA) having an IC_50_ value of 103.16 ± 0.56 μg/mL (Figure 1a).

#### 3.4.2 Hydroxyl radical scavenging assay

The hydroxyl radical scavenging activity of the Me‐BLL revealed appreciably scavenged hydroxyl radical produced from the decomposition of deoxyribose in the Fenton reaction (Figure 1b). The Me‐BLL showed the most effective hydroxyl radical scavenging activity with an IC_50_ value of 145.08 ± 6.62 μg/mL while reference standard catechin was 37.12 ± 3.59 μg/mL.

#### 3.4.3 Iron‐chelating effect

The iron‐chelating effects of the Me‐BLL were shown in Figure 1c. Me‐BLL showed the most significant iron chelation effect with an IC_50_ value of 95.14 ± 0.58 μg/mL while reference standard ascorbic acid (AA) revealed 120.06 ± 0.52 μg/mL (Figure 1c).

### 3.5 *Anxiolytic* *activity*

#### 3.5.1 Elevated plus maze test

**FIGURE 2:**
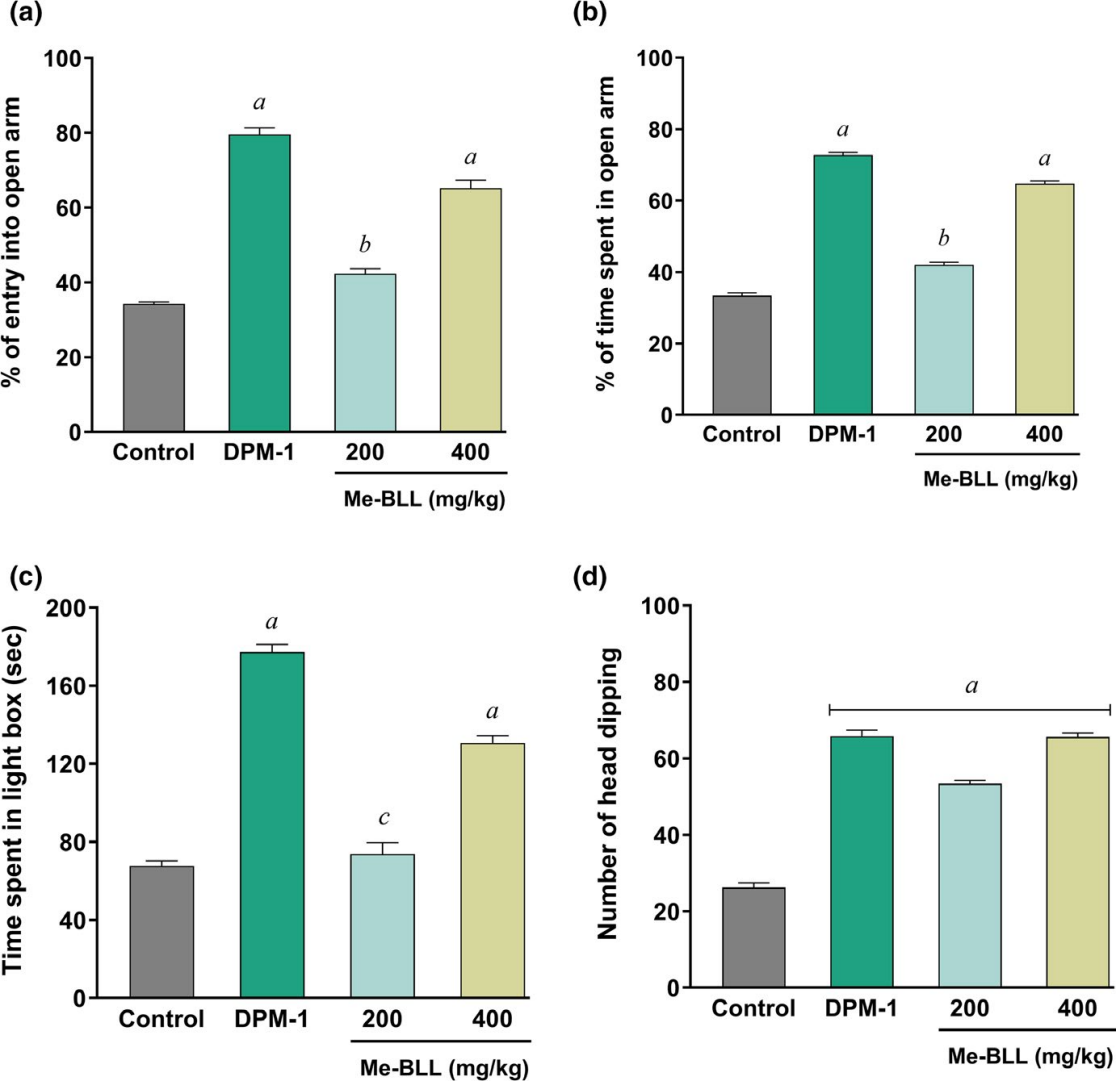
(a) Effects of Me‐BLL on %entry in the open arm in EPM test; (b) Effects of Me‐BLL on %time spent in the open arm in EPM test; (c) Effects of Me‐BLL on time spent in light box in LDB test; (d) Effects of Me‐BLL on the number of head dipping in HBT. All the values are expressed as mean ± *SEM*. ^c^ *p <0*.*05* and ^b^ *p <0*.*01*, significantly different from control. Me‐BLL = methanol extract of *Blumea lacera*; DPM‐1 = Diazepam 1 mg/kg; EPM = elevated plus maze; LDB = light and dark box; HBT = hole‐board test

In the EPM test, all the tested doses of Me‐BLL increased the entries into the open arms (Figure 2a). The values for 400 mg/kg (65.20 ± 2.10%) and reference drug diazepam (79.57 ± 1.84%) were noted statistically significant (*p <* .*01*) as compared to the control (34.21 ± 0.53%). Correspondingly, the values for a low dose at 200 mg/kg were also noted as significant (*p <* .*05*). Besides, all the tested doses demonstrated a dose‐dependent increase of time spent in open arms (Figure 2b).

#### 3.5.2 Light and dark box test

In the LDB test, all the tested doses demonstrated a dose‐dependent increase of time spent in the light compartment (Figure 2c). There was a notable increase in the duration of time (130.6 ± 3.72 s) for 400 mg/kg, into the light compartment, which was found significant (*p <* .*01*) compared to control (67.60 ± 2.67 s) while the value for reference drug was 177.2 ± 3.89 s (*p <* .*01*).

#### 3.5.3 Hole‐board test

In HBT, the oral administration of Me‐BLL in experimental animals has resulted in a significant number of head dipping (*p <* .*01*) at the doses of 200 mg/kg (53.40 ± 0.81) and 400 mg/kg (65.60 ± 1.02) in comparison to the control. Interestingly, the finding for 400 mg/kg dose was found almost similar to that of the standard drug diazepam (65.80 ± 1.62, *p <* .*01*) (Figure 2d).

### 3.6 *Antidepressant* *activity*

**FIGURE 3:**
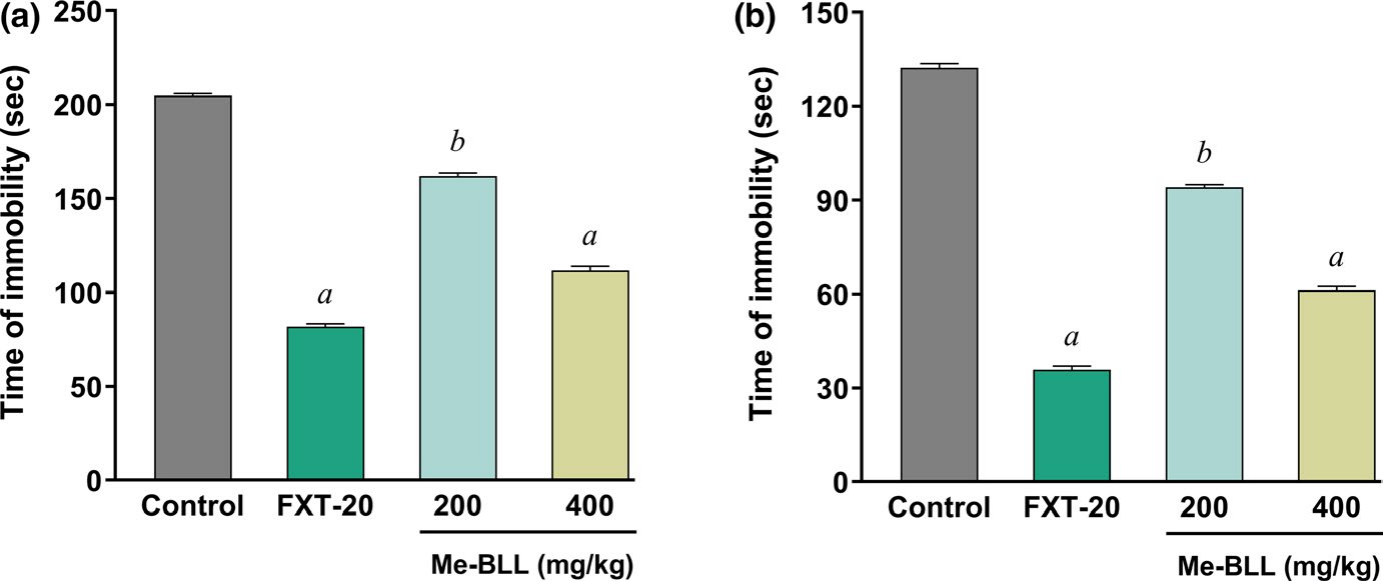
(a) Effects of Me‐BLL on times of immobility in TST; (b) Effects of Me‐BLL on times of immobility in FST. All the values are expressed as mean ± *SEM*. ^c^ *p <0*.*05* and ^b^ *p <0*.*01*, significantly different from control. Me‐BLL = methanol extract of *Blumea lacera*; FXT‐20 = Fluoxetin HCl 20 mg/kg; TST = tail suspension test; FST = forced swimming test

The effects of treatment with Me‐BLL on the duration of immobility times for TST and FST were presented in Figure 3a,b, respectively. In TST, times of immobility were decreased significantly to 161.8 ± 1.77 s (*p <* .*05*) and 111.8 ± 2.31 s (*p <* .*01*) for the doses of 200 and 400 mg/kg, respectively as compared to the control (204.8 ± 1.15 s). Additionally, the reference drug also exhibited a significant (*p <* .*01*) immobility time of 81.80 ± 1.65 s (Figure 3a). In FST, immobility times were decreased to 94.20 ± 0.86 and 61.20 ± 1.28 s with the respective two doses. Besides, the reference drug fluoxetine HCl exhibited an immobility time of 35.80 ± 1.15 s. The values were noted statistically significant (*p <* .*05*, *p <* .*01*) as compared to the control (132.21 ± 1.39 s) (Figure 3b).

### 3.7 *Computational* *studies*

#### 3.7.1 Molecular docking study of antioxidant activity

**FIGURE 4:**
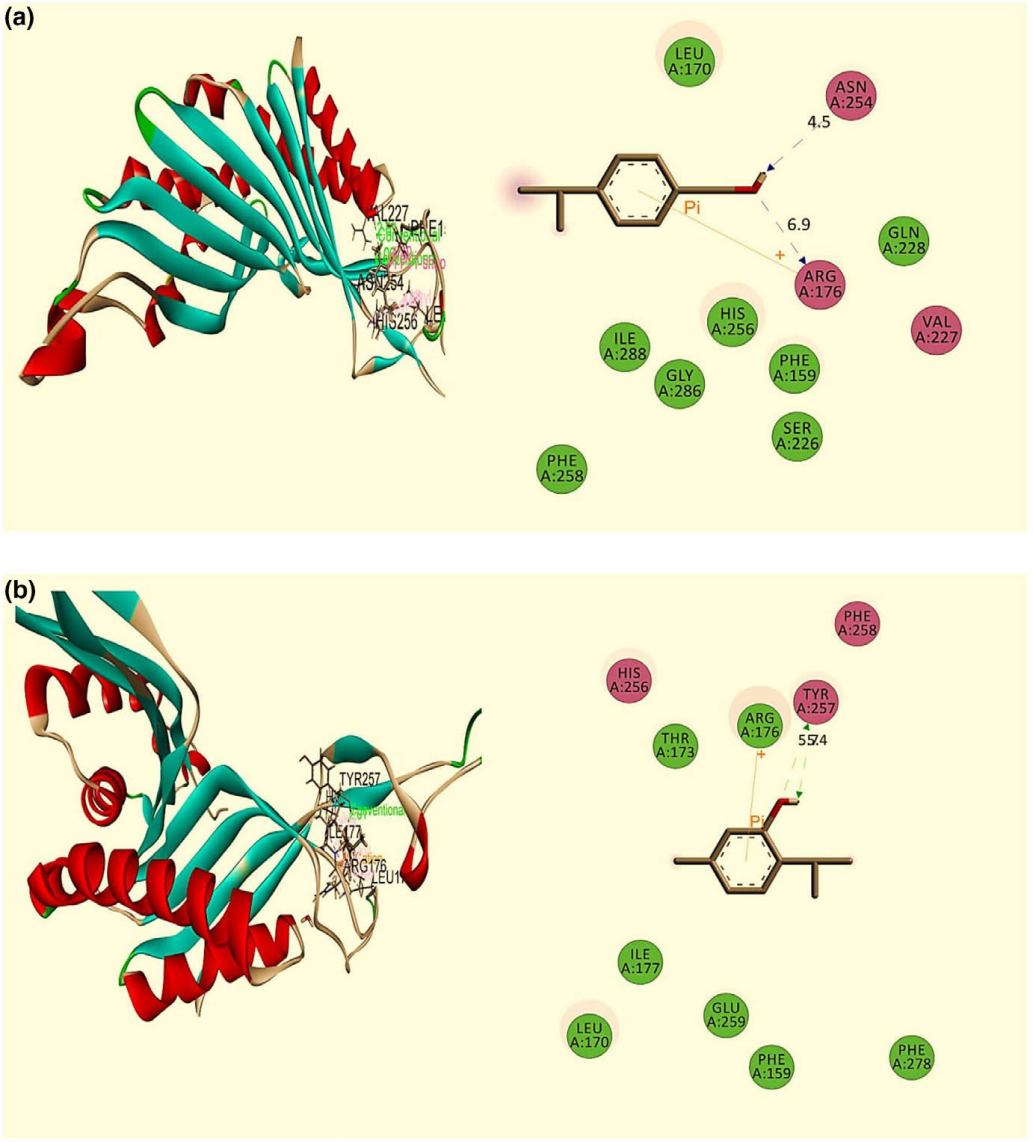
Best ranked poses and 2D interactions of (a) Cuminol (b) Thymol with urate oxidase (Uox) enzyme receptor (PDB: 1R4U) for antioxidant activity

The antioxidant molecular docking study of fifteen selected compounds was found to interact against the urate oxidase (Uox) enzyme (PDB: 1R4U) (Table 4). Among fifteen compounds, cuminol and thymol exhibited the highest binding affinities with a docking score of −4.67 kcal/mol and −4.617 kcal/mol, respectively, whereas the standard drug ascorbic acid showed −4.512 kcal/mol. The order of docking scores for antioxidant activities is noted as: cuminol >thymol > vitamin E > 13‐Docosenamide, (Z)‐ > squalene >stigmasterol > stigmast‐5‐en‐3 beta‐ol >gamma‐sitosterol >phytol > 9‐octadecenamide, (Z)‐ > linoleic acid, methyl ester >neophytadiene > 9‐Octadecenoic acid, methyl ester, (E)‐ > hexadecanoic acid, methyl ester. The best‐docked compound cuminol interacted with the Uox enzyme (PDB: 1R4U) by two H‐bond at Asn 254 (2.06 Å), Val 227 (2.61 Å), two Alkyl interaction at Leu 170 (4.69, 4.62 Å), and one Pi‐Alkyl interaction at His 256 (3.86 Å) (Figure 4). The thymol interacted by one H‐bond: Tyr 257 (2.01 Å); two Pi‐Alkyl interaction: His 256 (4.56 Å), Arg 176 (4.92 Å), and three Alkyl interaction: Arg 176 (3.94 Å), Leu 170 (4.36 Å); Ile 177 (4.38 Å) (Figure 4). The 12 compounds also revealed a good docking score with the Uox enzyme (PDB: 1R4 U) (Figure S2‐S5).

**TABLE 4:** Docking score of the selected compound from GC‐MS data of Me‐BLL

Compounds	PubChem ID	1R4 U	4UUJ	5I6X
Cuminol	325	**−4.67**	**−4.602**	−6.507
Thymol	6,989	−4.617	−4.361	−6.051
Neophytadiene	10,446	+0.922	+0.015	−2.457
Hexadecanoic acid, methyl ester	8,181	+1.39	+1.091	−0.951
Linoleic acid, methyl ester	5,284,421	+0.743	‐‐	−2.447
9‐Octadecenoic acid, methyl ester, (E)‐	5,280,590	+1.241	+0.906	−2.074
Phytol	5,280,435	−0.461	−0.949	−2.818
Stigmast‐5‐en‐3.beta.‐ol	222,284	−2.354	−2.259	**−7.947**
Gamma‐Sitosterol	457,801	−2.105	−3.165	−7.938
9‐Octadecenamide, (Z)‐	5,283,387	+0.075	+0.421	−3.126
Widdrol	94,334	‐‐	‐‐	‐‐
13‐Docosenamide, (Z)‐	5,365,371	−2.899	−3.349	−6.4
Squalene	638,072	−2.651	−4.347	−5.982
Vitamin E	14,985	−3.171	−3.764	−7.2
Stigmasterol	5,280,794	−2.5	−2.403	‐‐
Ascorbic acid/Diazepam/Fluoxetine	54670067/3016/3386	−4.512	−3.035	−9.07

Note

Docking scores in kcal/mol; bold text indicates the highest score.

#### 3.7.2 Molecular docking study of anxiolytic activity

**FIGURE 5:**
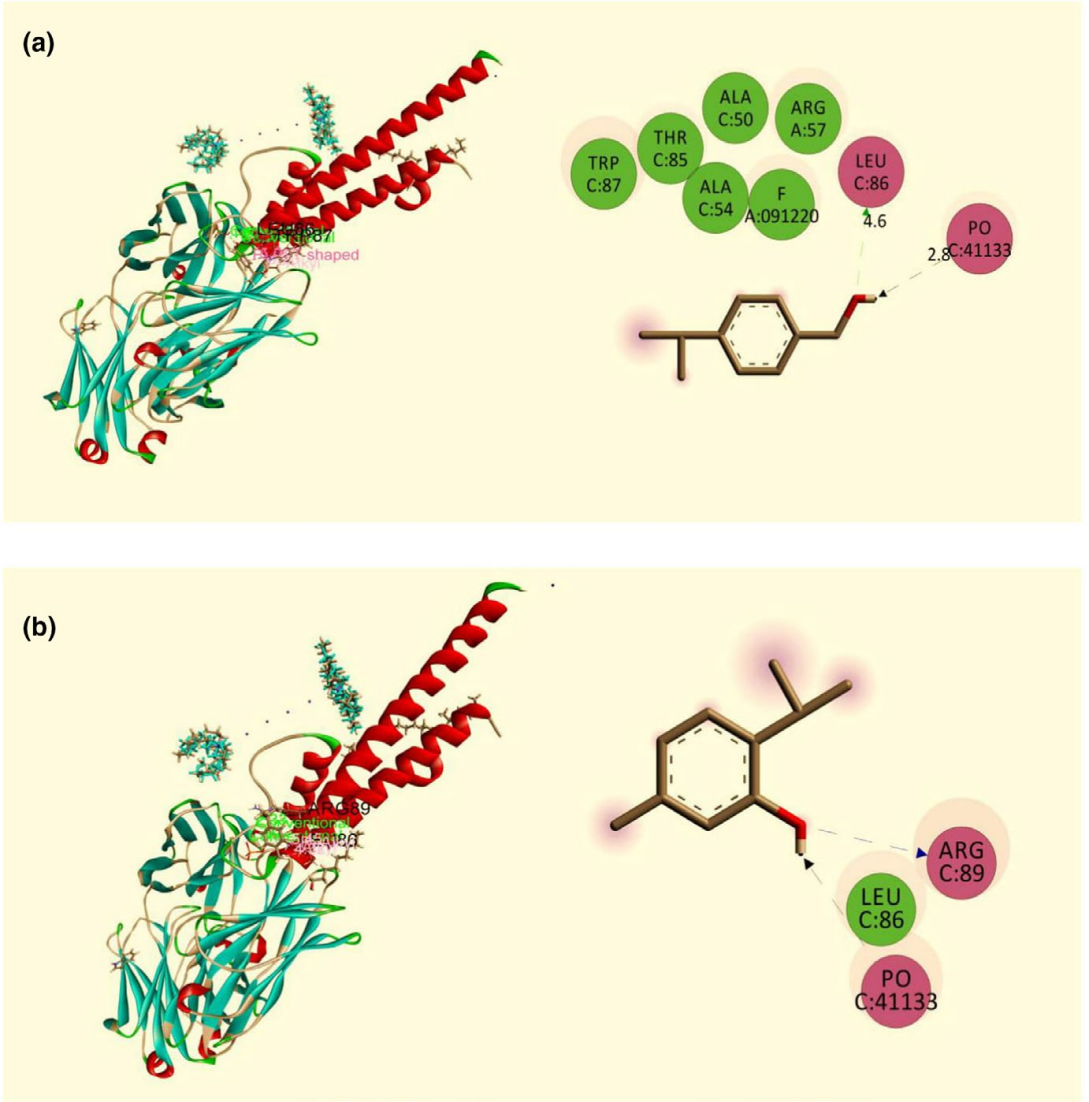
Best ranked poses and 2D interactions of (a) Cuminol (b) Thymol with potassium channel receptor (PDB: 4UUJ) for anxiolytic activity

The anxiolytic molecular docking study of fifteen selected compounds was noted to interact against the potassium channel (PDB: 4UUJ) (Table 4). From fifteen compounds, cuminol, thymol, and squalene exhibited the highest binding affinity, with a docking score of −4.602 kcal/mol, −4.361 kcal/mol, and −4.347 kcal/mol, respectively. In contrast, the standard drug diazepam showed −3.035 kcal/mol. The order of docking score for anxiolytic activities was noted as: cuminol >thymol > squalene >vitamin E > 13‐Docosenamide, (Z)‐ > gamma‐sitosterol >stigmasterol > stigmast‐5‐en‐3 beta‐ol >phytol > neophytadiene >9‐Octadecenamide, (Z)‐ > 9‐Octadecenoic acid, methyl ester, (E)‐ > hexadecanoic acid, methyl ester. The linoleic acid, methyl ester, and widdrol did not pose any interaction with the receptor. The best‐docked compound cuminol interacted with the potassium channel (PDB: 4UUJ) receptor by two H‐bond at Leu 86 (2.08 Å), PO 41,133 (1.67 Å), one Pi‐Pi interaction at Trp 87 (5.50 Å), and one Pi‐Alkyl interaction at Trp 87 (4.23 Å) (Figure 5). The thymol interacted by one H‐bond: Arg 89(2.33 Å); Pi‐alkyl and alkyl interaction: Leu 86 (5.45 Å, 4.32 Å) (Figure 5). The other 11 compounds also revealed a good docking score with the potassium channel (PDB: 4UUJ) receptor (Figure S6–S9).

#### 3.7.3 Molecular docking study of antidepressant activity

**FIGURE 6:**
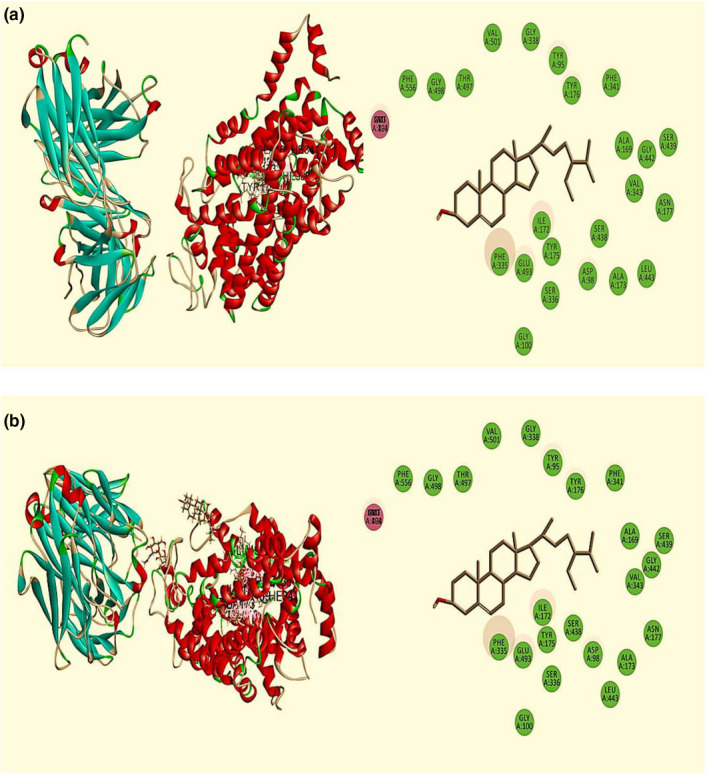
Best ranked poses and 2D interactions of (a) Stigmast‐5‐en‐3.beta.‐ol (b) Gamma‐Sitosterol with human serotonin receptor (PDB: 5I6X) for antidepressant activity

The molecular docking study of antidepressant activity is summarized in Table 4. From fifteen compounds, stigmast‐5‐en‐3.beta.‐ol and hexadecanoic acid, methyl ester demonstrated the highest and lowermost binding affinity against human serotonin receptor (PDB: 5I6X) with a docking score of −7.947 kcal/mol and −0.951 kcal/mol, respectively, whereas the standard drug fluoxetine HCl showed −9.07 kcal/mol. The order of docking score for antidepressant activities was noted as: Stigmast‐5‐en‐3.beta.‐ol >gamma‐sitosterol >vitamin E > cuminol >13‐Docosenamide, (Z)‐ > thymol >squalene > 9‐Octadecenamide, (Z)‐ > phytol >neophytadiene > linoleic acid, methyl ester >9‐Octadecenoic acid, methyl ester, (E)‐ > hexadecanoic acid, methyl ester. Stigmasterol and widdrol did not pose any interaction with the receptor. The best‐docked compound stigmast‐5‐en‐3.beta.‐ol interacted with the human serotonin receptor (PDB: 5I6X) receptor by two H‐bond at Arg 104 (2.71 Å), Glu 494 (2.63 Å), six alkyl interaction at Ile 172, Ala 173, Ala 169, and six Pi‐Aalkyl interaction at Phe 335 (3.33 Å, 4.81 Å, 4.95 Å), Phe 341 (3.43 Å), Tyr 176 (3.58, 4.45 Å) (Figure 6). Another best compound, gamma‐sitosterol, also exhibited higher binding affinity (Figure 6). The other 11 compounds also revealed a good docking score with the human serotonin receptor (PDB: 5I6X) receptor (Figure S10–S13).

#### 3.7.4 Pharmacokinetic and toxicological properties

The ADME/T properties of selected eight compounds were assessed by the Lipinski's rule of five and Veber's rules. Here, cuminol and thymol followed all the rules, whereas six other compounds violated one rule (Table 5). As all the compounds followed Lipinski's and Veber's rules except one rule, all the compounds can be predicted with good oral bioavailability. Besides, the toxicological properties of eight compounds predicted non‐ames toxicity, non‐carcinogenicity, weak rat toxicity except for cuminol and squalene (Table 6).

**TABLE 5:** Pharmacokinetic properties of the selected bioactive secondary metabolites in Me‐BLL

Compounds	Lipinski Rules				Lipinski's violations (≤1)	Veber Rules	
	MW (<500)	HBA (<10)	HBD (<5)	Log P (≤5)		Nrb (≤10)	TPSA (≤140 Å^2^)
Cuminol	150.22	1	1	2.34	0	2	20.23
Thymol	150.22	1	1	3.30	0	1	20.23
Stigmast‐5‐en‐3.beta.‐ol	414.71	1	1	9.34	1	6	20.23
Gamma‐Sitosterol	414.71	1	1	9.34	1	6	20.23
13‐Docosenamide, (Z)‐	337.58	1	1	9.16	1	19	43.09
Squalene	410.72	0	0	11.58	1	15	0
Vitamin E	430.71	2	1	10.7	1	12	29.46
Stigmasterol	412.69	1	1	8.56	1	5	20.23

Abbreviations: HBA, hydrogen bond acceptor; HBD, hydrogen bond donor; Log P, lipophilicity; MW, molecular weight (g/mol); nRB, number of rotatable bond; TPSA, topological polar surface area.

**TABLE 6:** Toxicological properties of the selected bioactive secondary metabolites in Me‐BLL

Compounds	Ames toxicity	Carcinogenicity	Acute oral toxicity	Weak rat acute toxicity
Cuminol	NAT	C	III	2.1367
Thymol	NAT	NC	III	2.2996
Stigmast−5‐en−3.beta.‐ol	NAT	NC	I	2.6561
Gamma.‐Sitosterol	NAT	NC	I	2.6561
13‐Docosenamide, (Z)‐	NAT	NC	III	2.0712
Squalene	NAT	C	III	1.5057
Vitamin E	NAT	NC	III	2.1598
Stigmasterol	NAT	NC	I	2.6561

Note

Category‐I (LD50 ≤ 50 mg/kg) and Category‐III (500 mg/kg <LD50 < 5,000 mg/kg).

Abbreviations: C, carcinogenic; NAT, non‐ames toxic; NC, non‐carcinogenic.

## 4 DISCUSSIONS

Herbal medicines have been used in traditional therapies around the world for many disorders. Dietary medicinal herbs are superior antioxidant reservoirs that are safe for long‐term intake (He et al., 2018). Besides, herbal medicine is turning into a feasible alternative treatment over the financially accessible synthetic drugs on neuropsychiatric management/treatment because of the lower cost, availability, and little or no adverse effects of herbal medicines (Nissen, 2010). Many well‐recognized herbal products have illustrated neuropharmacological properties while mitigating anxiety and depression (Sarwar et al., 2018). Animal behavior tools are inevitable tools for the determination and development of top‐notch anxiolytic and antidepressant drugs. In this present study, Swiss albino mice were used as an experimental model to investigate the neuropharmacological potentials of Me‐BLL in addition to its phytochemical profiling.

Phytochemical profiling of this research demonstrated a wide variety of phytochemicals, including alkaloids, carbohydrates, phenols, flavonoids, proteins, and amino acids through qualitative phytochemical screening GC‐MS analysis. The quantitative phytochemical analysis also revealed a prominent amount of phenolic and flavonoid contents in Me‐BLL. The outcomes suggest that the Me‐BLL can decline the risk of several neurological disorders, including anxiety and depression, by eliciting the antioxidative activities to prevent ROS or cellular damage. Recent studies revealed that flavonoids act as free radical scavengers of many oxidizing species (Reza et al., 2018) and found effective in the central nervous system (Matias et al., 2016). However, the antioxidant activity of Me‐BLL was evaluated employing DPPH, hydroxyl radical scavenging assay, and iron‐chelating activities were done following established protocol. The overall results showed promising antioxidative effects of Me‐BLL. Earlier studies suggested that the polyphenolic compounds are potent scavenger of free radicles and ROS, and their strong antioxidant activity is ascribed to the presence of ortho hydroxyl grouping, which blocks the free radical reaction and generates a hydrogen atom transfer reaction (Granato et al., 2018; Rahman et al., 2020). Moreover, a study demonstrated an elevated level of ROS produces many known mechanisms like mitochondrial deregulations, lipid cellular structures, neurotransmitter deregulations, and cellular respiration, which correlates with anxiety and depression (Rammal et al., 2010). In this regard, antioxidant attenuates anxiety and depression, and inhibition of ROS formation is intervened through redox‐related signaling pathways. The study assumes that Me‐BLL has potential antioxidant properties as significant DPPH, H_2_O_2_ scavenging, and iron‐chelating activities were manifested, finally, may alleviate neuropsychiatric disorders. In this context, a neuropharmacological insight of the Me‐BLL was further evaluated through an established protocol.

The use of the EPM test is considered a standard technique to analyze anxiety‐like behavior. Test samples with anxiolytic properties diminish the removal of open arms and induce test animals to spend more time in open arms, whereas anxiogenic halt open arm investigation reduces both the number of spent time into the open arms (Griebel et al., 2000). Our results revealed that the administration of Me‐BLL at different doses showed a tendency to expand the time spent in open arms, an indicator of diminished anxiety. Like EPM, in the LDB test, an expansion of the number of times spent in the light box indicates the anxiolytic activity. The administration of Me‐BLL increased the amount of time spent in open arms, the number of head dips, and the amount of time spent in the light box, all of which indicated a decrease in anxiety‐like disorder. The anxiolytic effects were further evaluated by the hole‐board test (HBT). The number of head dipping in the HBT gives an exploratory pattern, and the expansion of the number of head dipping demonstrates a sign of anxiolytic activity. There was an increase in the number of head dipping when treated with Me‐BLL, which is a sign of anxiolytic activity (Casarrubea et al., 2015). To evaluate the antidepressant activity, observation of the immobile time of the test animals was analyzed through forced swimming and tail suspension test (FST and TST). From our investigation, it is evident that the administration of Me‐BLL significantly decreased the immobility time. It is credible that Me‐BLL may act by potentiating GABA restraint in the CNS through film hyperpolarization since GABA is the major inhibitory synapse in the CNS, and it prompts a decrease in the terminating rate of basic neurons in the brain. This restraint due to the direct actuation of the GABA receptor by the secondary metabolites presents in Me‐BLL.

In computer‐aided drug design, in silico molecular docking study is a pivotal tool that predicts the binding activity of compounds against particular proteins (Hossen et al., 2021; S. Khan et al., 2019). Besides, the possible molecular mechanism of actions of different pharmacological activities is determined comprehensively through molecular docking. Therefore, molecular docking was performed to correlate with the present neuropharmacological findings, finally, to understand the better molecular mechanism. In this experiment, fifteen major bioactive compounds of Me‐BLL interacted against three target receptors or enzymes, namely, urate oxidase (Uox) enzyme receptor (PDB: 1R4U), potassium channel receptor (PDB: 4UUJ), and human serotonin receptor (PDB: 5I6X). Among these, eight compounds, namely stigmast‐5‐en‐3.beta.‐ol, gamma‐sitosterol, vitamin E, cuminol, 13‐Docosenamide (Z)‐, thymol, squalene, and phytol, were found potential that docked against the receptors as mentioned above for antioxidant, anxiolytic, and antidepressant effects, respectively. Thus, the biological activities of Me‐BLL might be delineated by the presence of these bioactive metabolites having remarkable docking scores.

To determine ADME/T and toxicological properties, eight compounds have been selected based on the highest scores in the molecular docking study against the mentioned receptors. The selected compounds are cuminol, thymol, stigmast‐5‐en‐3.beta.‐ol, gamma‐sitosterol, 13‐Docosenamide (Z)‐, squalene, vitamin E, and stigmasterol, respectively. According to the Lipinski's rule of five, administration of the oral drug should have a molecular weight <500 amu, hydrogen bond acceptor sites <10, hydrogen bond donor sites <5, and lipophilicity value, LogP ≤5 whereas Veber et al. (Veber et al., 2002) suggested that a compound/drug should have the number of rotatable bonds (nRB) ≤10 and topological polar surface area (TPSA) value ≤140 Å2. If any drug/compound violates all of these rules, it will not be considered good oral bioavailability. The present investigation exhibited that most of the compounds violated one rule, which indicates good oral bioavailability. Besides, the toxicological analysis demonstrated that none of the compounds posed a risk of Ames toxicity, acute oral toxicity, and weak rat acute toxicity and, therefore, can be considered safe.

## 5 CONCLUSION

The present study revealed that this edible plant has significant anxiolytic and antidepressant effects as well as antioxidant potential. Moreover, different bioactive compounds of Me‐BLL unveiled a promising avenue with a binding attraction toward different proteins in molecular docking analysis. It is noteworthy that the selected active compounds have elucidated their drug‐like characteristics and safeness in ADME/T and toxicology studies. Therefore, it can be considered as an alternative food product for neuropsychiatric treatment. Further mechanistic research followed by the dose‐response study is strongly recommended to elicit the neuroprotective activities of this promising plant.

## 6 STUDIES INVOLVING ANIMAL SUBJECTS

The experimental mice were managed according to the “Guide for the Care and Use of Laboratory Animals,” eight edition, USA. Animals were handled and maintained according to the recommended protocol of the Institutional Animal Ethics Committee, Department of Pharmacy, International Islamic University Chittagong, Bangladesh (reference number: P&D‐147/13‐19).

## CONFLICT OF INTEREST

The authors declared that they have no conflicts of interest in this work.

## AUTHOR CONTRIBUTION

**Md. Amjad Hossen:** Formal analysis (supporting); Investigation (lead); Methodology (supporting); Software (supporting); Writing‐original draft (lead). **A. S. M. Ali Reza:** Conceptualization (lead); Formal analysis (supporting); Funding acquisition (lead); Methodology (supporting); Project administration (lead); Supervision (lead); Writing‐review & editing (supporting). **Md. Badrul Amin:** Data curation (equal); Investigation (supporting); Software (equal); Writing‐original draft (supporting). **Mst. Samima Nasrin:** Investigation (supporting); Methodology (supporting); Resources (supporting); Visualization (supporting). **Tawhidul Amin Khan:** Investigation (supporting); Methodology (supporting); Validation (supporting). **Md. Habibur Rahman Rajib** **:** Investigation (supporting); Methodology (supporting); Validation (supporting). **Abu Montakim Tareq:** Formal analysis (supporting); Software (supporting). **Md. Anwarul Haque:** Conceptualization (supporting); Data curation (supporting); Resources (supporting); Visualization (supporting). **Md. Atiar Rahman:** Conceptualization (supporting); Methodology (supporting); Resources (supporting); Visualization (supporting); Writing‐review & editing (supporting). **Md. Areeful Haque:** Conceptualization (supporting); Data curation (lead); Formal analysis (lead); Software (supporting); Supervision (supporting); Writing‐original draft (supporting); Writing‐review & editing (lead).

## Supporting information

Supplementary Material

## Data Availability

The data that support the findings of this study are available on request from the corresponding author.
