# Supplementary material for: Bioactive metabolites of Blumea lacera attenuate anxiety and depression in rodents and computer‐aided model
Source: Food Sci Nutr. 2021 May 31;9(7):3836–51. doi: 10.1002/fsn3.2362 (PMC8269660; doi:10.1002/fsn3.2362)
Supplement: Supplementary file 1 — Supplementary Material [file FSN3-9-3836-s001.docx]

**Supplementary materials**

**Figures**

**
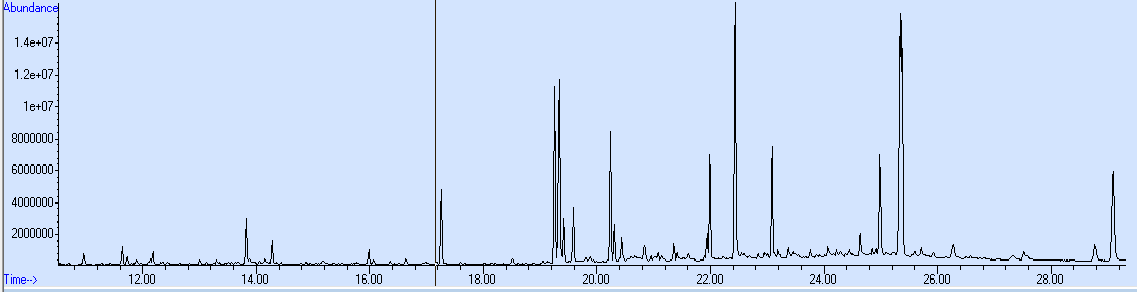
**

**Figure S1.** Total ionic chromatogram (TIC) of Me-BLL by GC-MS.

**
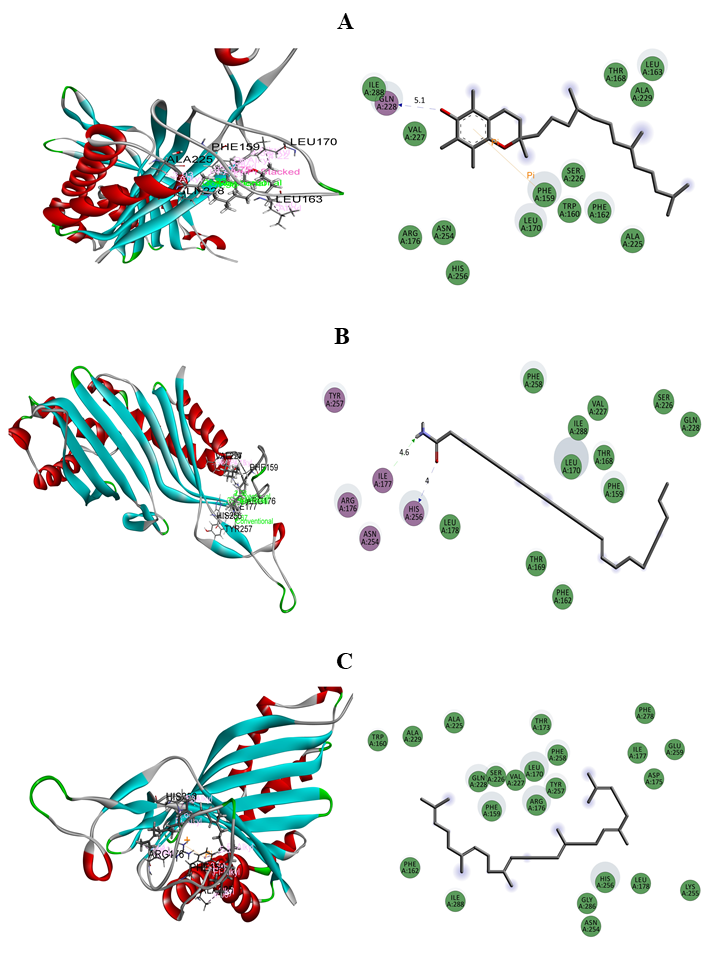
**

**Figure S3.**

**Figure S2.** Best ranked poses and 2D interactions of **(A)** Vitamin E **(B)** 13-Docosenamide, (Z)- **(C)** Squalene with urate oxidase (Uox) enzyme receptor (PDB: 1R4U) for antioxidant activity.

**
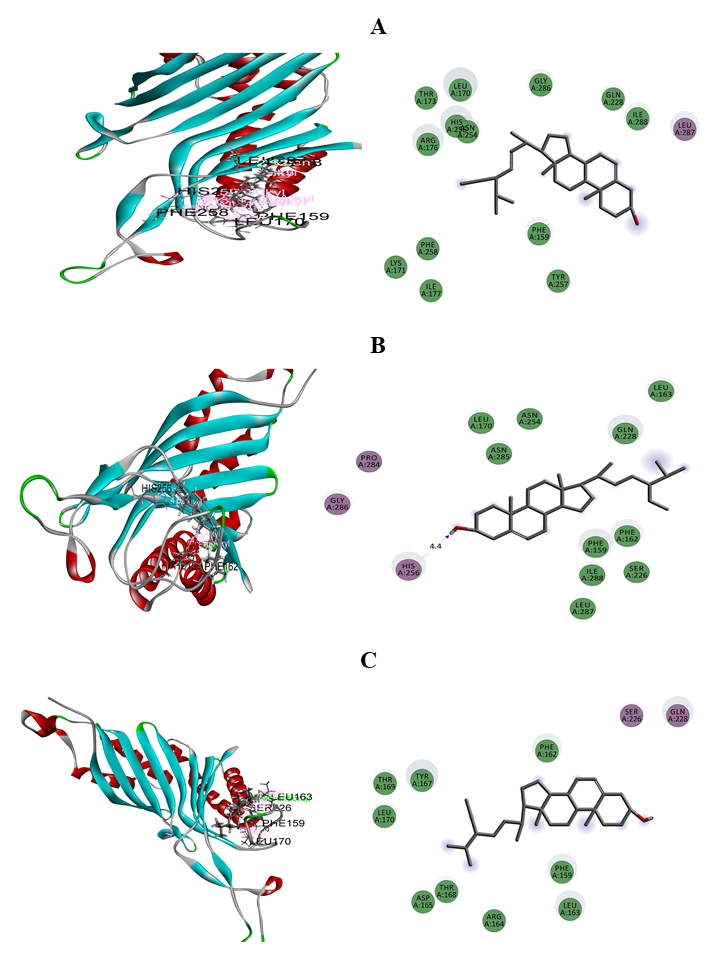
**

**Figure S3.** Best ranked poses and 2D interactions of **(A)** Stigmasterol **(B)** Stigmast-5-en-3 beta-ol **(C)** gamma-sitosterol with urate oxidase (Uox) enzyme receptor (PDB: 1R4U) for antioxidant activity.

**
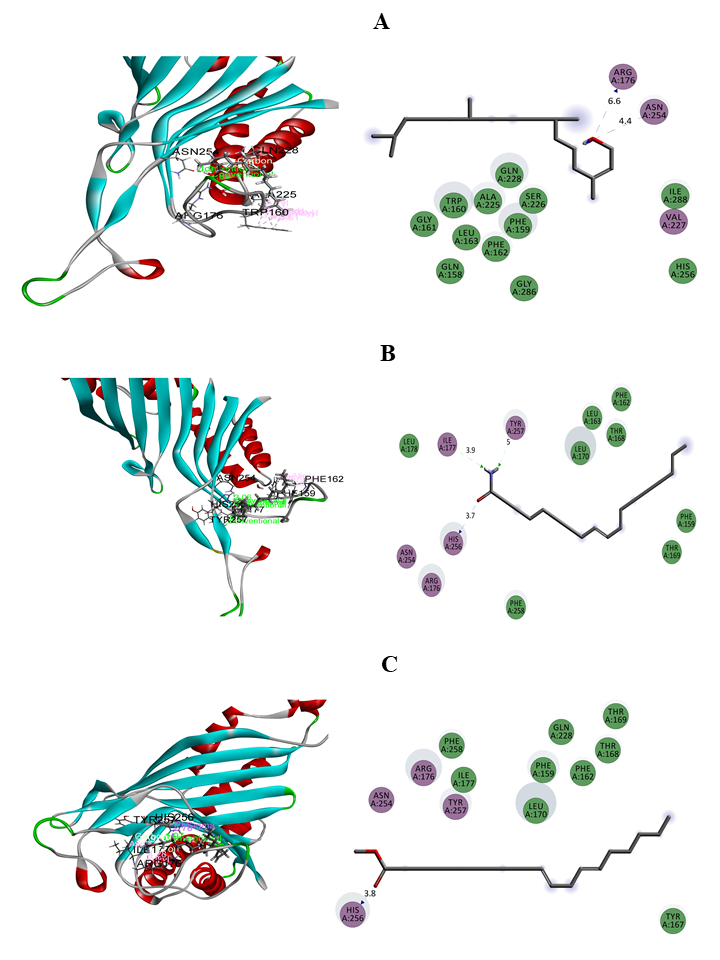
**

**Figure S4.** Best ranked poses and 2D interactions of **(A)** Phytol **(B)** 9-Octadecenamide, (Z)- **(C)** Linoleic acid, methyl ester with urate oxidase (Uox) enzyme receptor (PDB: 1R4U) for antioxidant activity.

**Figure S 3.** Best ranked poses and 2D interactions of **(A)** Phytol **(B)** 9-Octadecenamide, (Z)- **(C)** Linoleic acid, methyl ester with urate oxidase (Uox) enzyme receptor (PDB: 1R4U) for antioxidant activity.

**Figure S 3.** Best ranked poses and 2D interactions of **(A)** Phytol **(B)** 9-Octadecenamide, (Z)- **(C)** Linoleic acid, methyl ester with urate oxidase (Uox) enzyme receptor (PDB: 1R4U) for antioxidant activity.

**
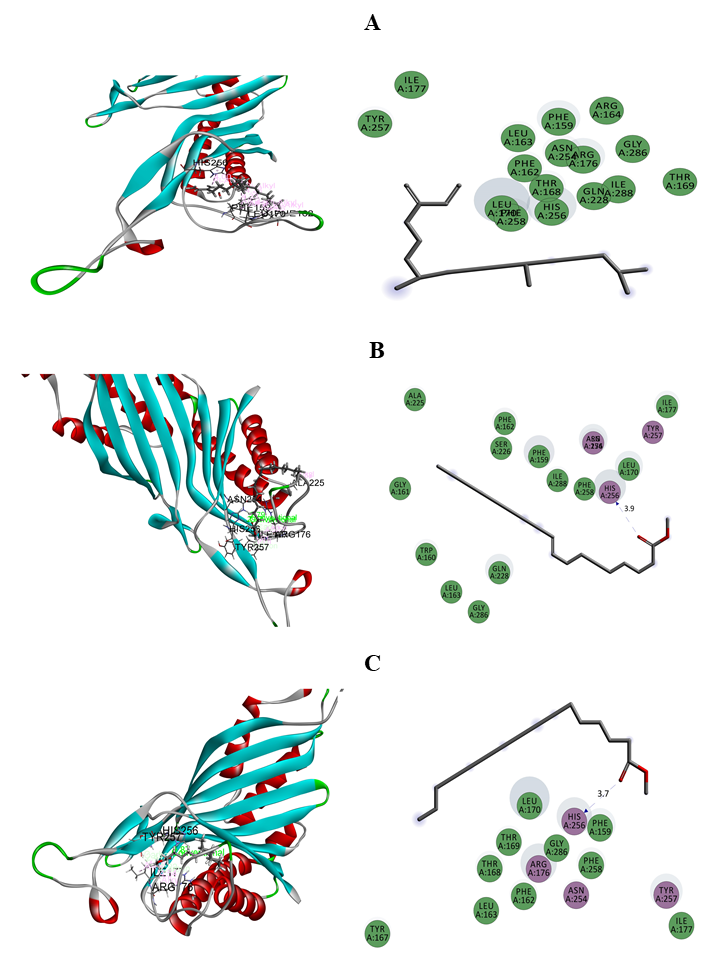
**

**Figure S5.** Best ranked poses and 2D interactions of **(A)** Neophytadiene **(B)** 9-Octadecenoic acid, methyl ester, (E)- **(C)** Hexadecanoic acid, methyl ester with urate oxidase (Uox) enzyme receptor (PDB: 1R4U) for antioxidant activity.

**Figure S 4.** Best ranked poses and 2D interactions of **(A)** Neophytadiene **(B)** 9-Octadecenoic acid, methyl ester, (E)- **(C)** Hexadecanoic acid, methyl ester with urate oxidase (Uox) enzyme receptor (PDB: 1R4U) for antioxidant activity.

**Figure S 4.** Best ranked poses and 2D interactions of **(A)** Neophytadiene **(B)** 9-Octadecenoic acid, methyl ester, (E)- **(C)** Hexadecanoic acid, methyl ester with urate oxidase (Uox) enzyme receptor (PDB: 1R4U) for antioxidant activity.

**
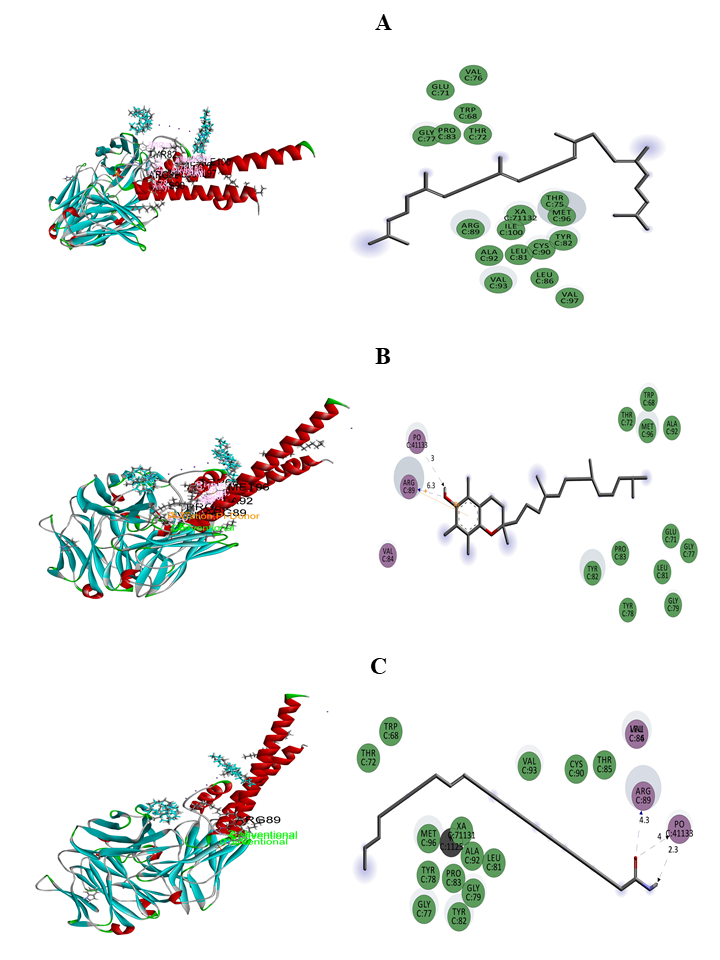
**

**Figure S6.** Best ranked poses and 2D interactions of **(A)** Squalene **(B)** Vitamin E **(C)** 13-Docosenamide, (Z)- with potassium channel receptor (PDB: 4UUJ) for anxiolytic activity.

**Figure S 5.** Best ranked poses and 2D interactions of **(A)** Squalene **(B)** Vitamin E **(C)** 13-Docosenamide, (Z)- with potassium channel receptor (PDB: 4UUJ) for anxiolytic activity.

**Figure S 5.** Best ranked poses and 2D interactions of **(A)** Squalene **(B)** Vitamin E **(C)** 13-Docosenamide, (Z)- with potassium channel receptor (PDB: 4UUJ) for anxiolytic activity.

**
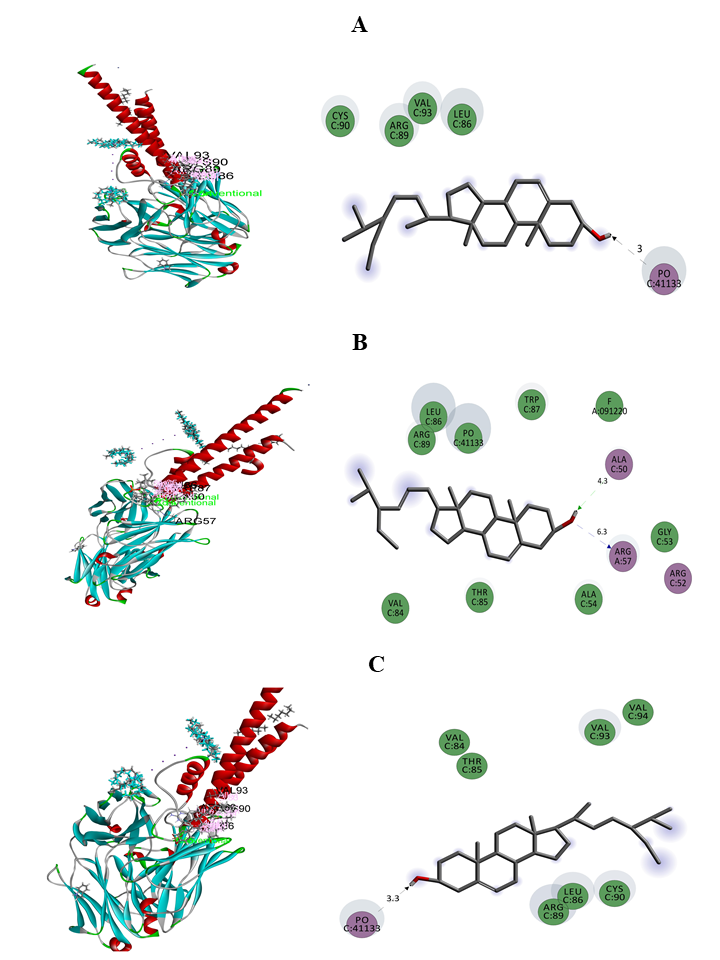
**

**Figure S10.**

**Figure S7**. Best ranked poses and 2D interactions of **(A)** gamma-sitosterol **(B)** Stigmasterol **(C)** Stigmast-5-en-3 beta-ol with potassium channel receptor (PDB: 4UUJ) for anxiolytic activity.

**
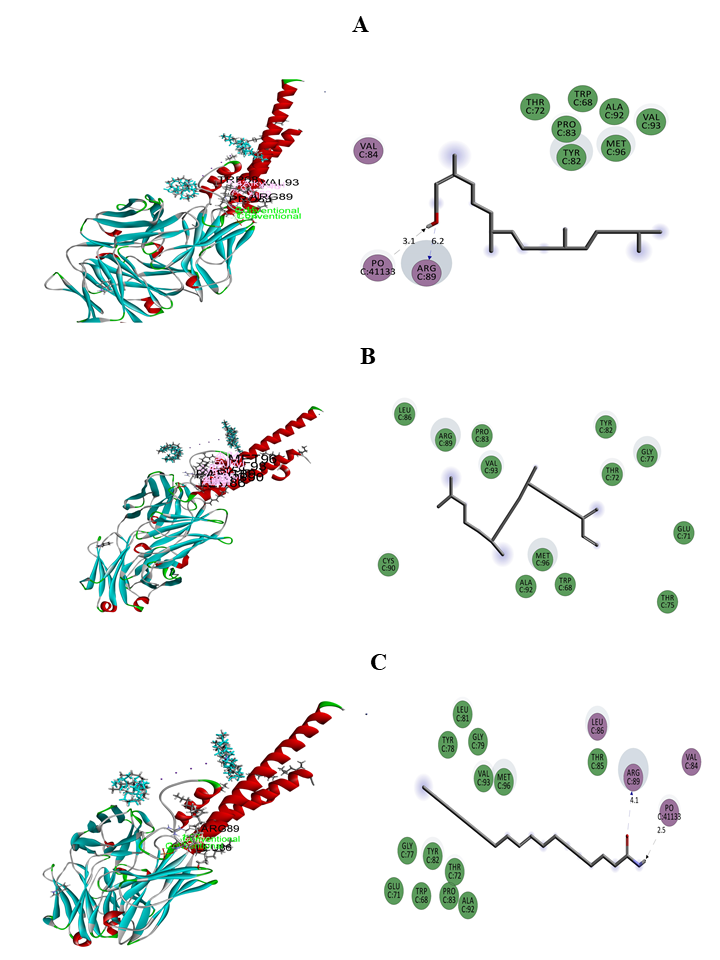
**

**Figure S8.** Best ranked poses and 2D interactions of **(A)** Phytol **(B)** Neophytadiene **(C)** 9-Octadecenamide, (Z)- with potassium channel receptor (PDB: 4UUJ) for anxiolytic activity.

**
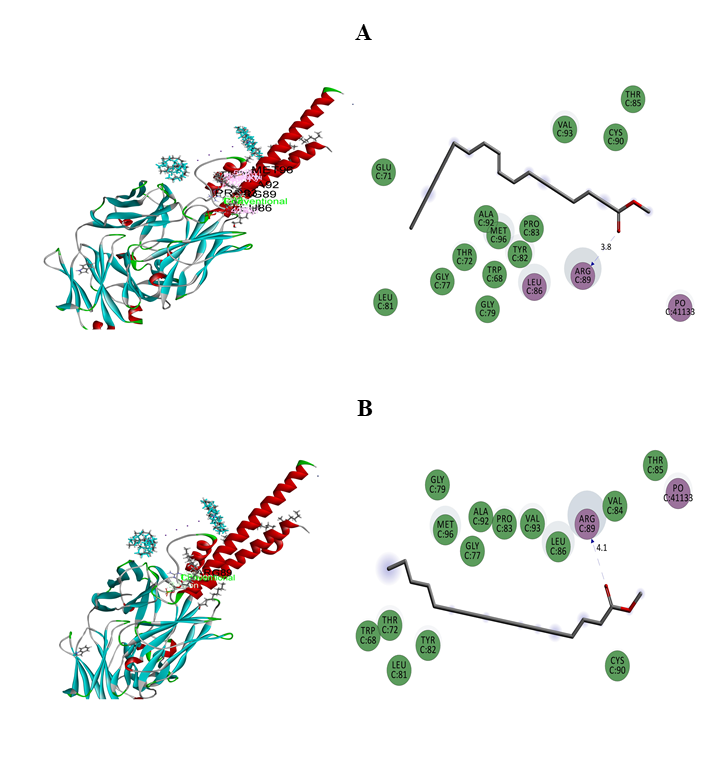
**

**Figure S9.** Best ranked poses and 2D interactions of **(A)** 9-Octadecenoic acid, methyl ester, (E)- **(B)** Hexadecanoic acid, methyl ester with potassium channel receptor (PDB: 4UUJ) for anxiolytic activity.

**
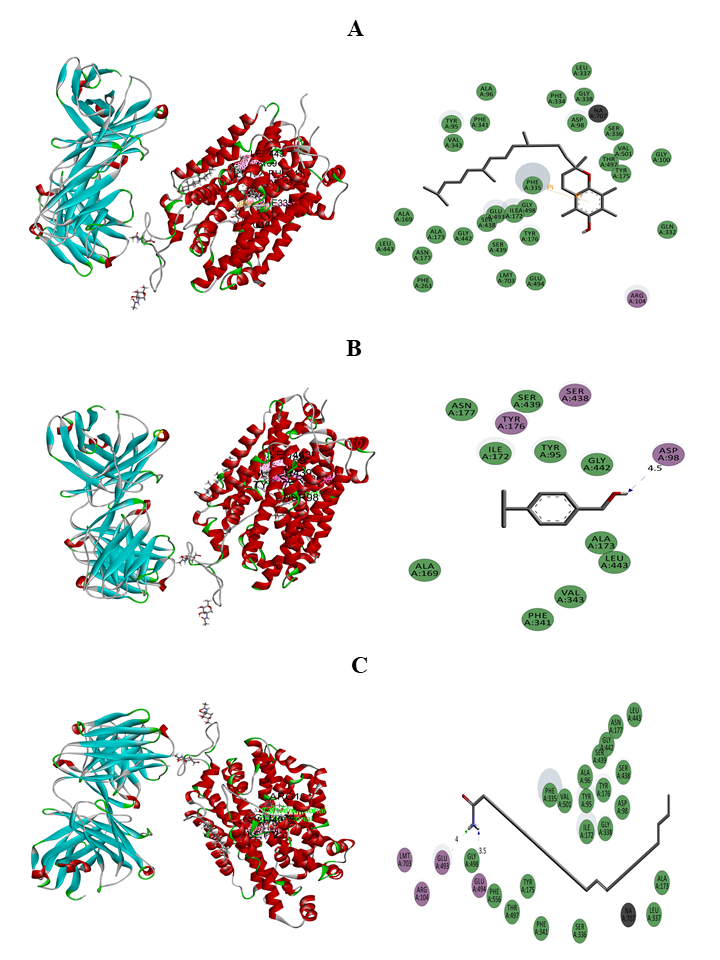
**

**Figure S10.** Best ranked poses and 2D interactions of **(A)** Vitamin E **(B)** cuminol **(C)** 13-Docosenamide, (Z)- with human serotonin receptor (PDB: 5I6X) for antidepressant activity.

**
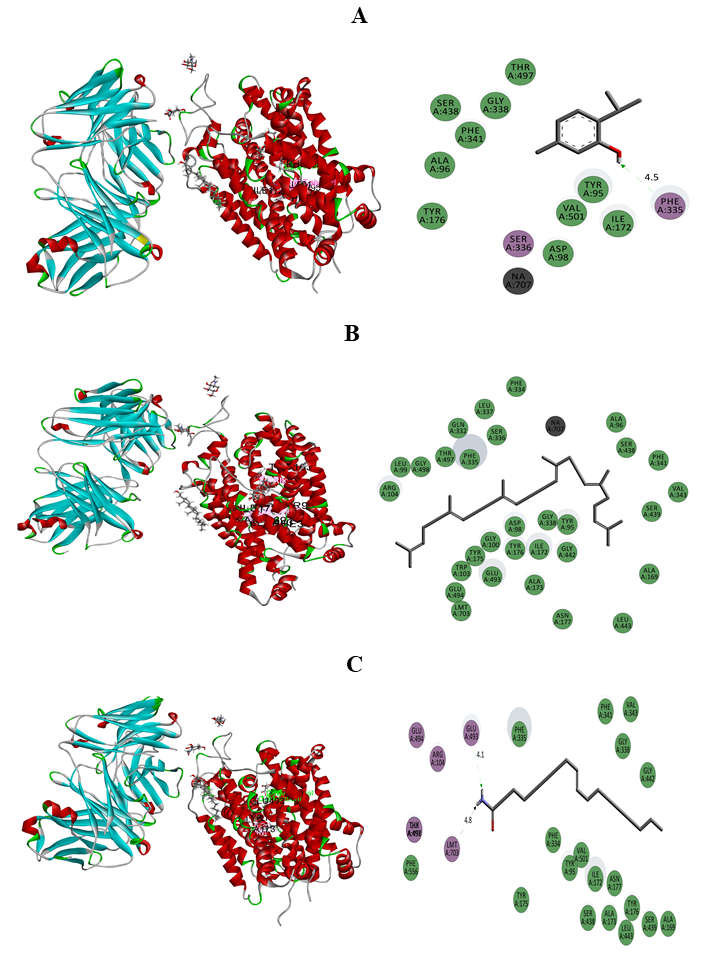
**

**Figure S11.** Best ranked poses and 2D interactions of **(A)** Thymol **(B)** Squalene **(C)** 9-Octadecenamide, (Z)-with human serotonin receptor (PDB: 5I6X) for antidepressant activity.

**Figure S 7.** Best ranked poses and 2D interactions of **(A)** Phytol **(B)** Neophytadiene **(C)** 9-Octadecenamide, (Z)- with potassium channel receptor (PDB: 4UUJ) for anxiolytic activity.

**Figure S 7.** Best ranked poses and 2D interactions of **(A)** Phytol **(B)** Neophytadiene **(C)** 9-Octadecenamide, (Z)- with potassium channel receptor (PDB: 4UUJ) for anxiolytic activity.

**
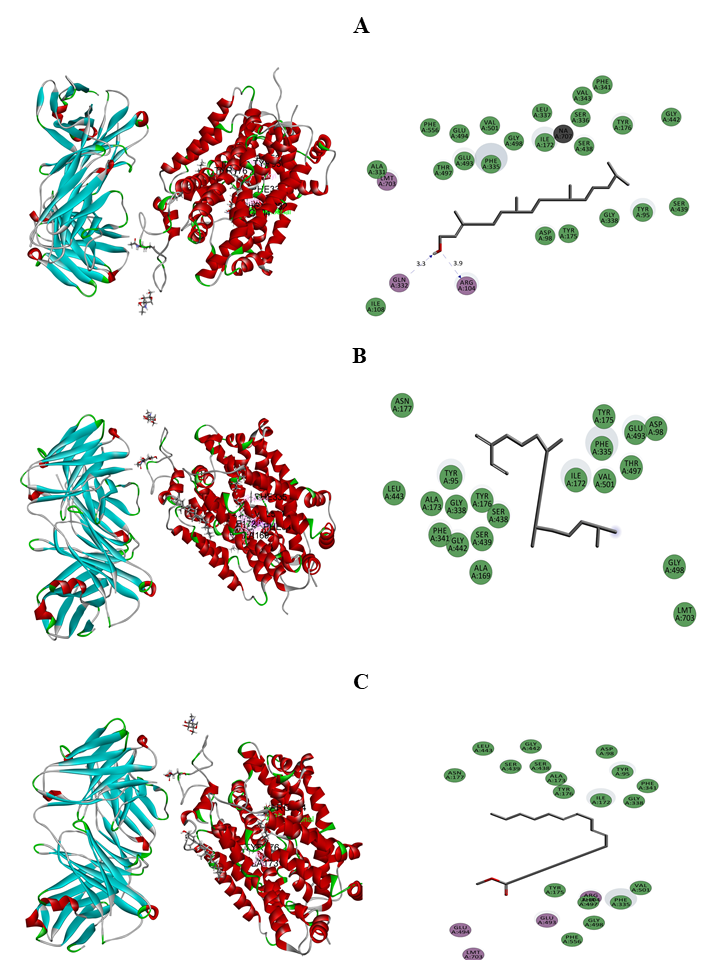
**

**Figure S12.** Best ranked poses and 2D interactions of **(A)** Phytol **(B)** Neophytadiene **(C)** Linoleic acid, methyl ester with human serotonin receptor (PDB: 5I6X) for antidepressant activity.

**
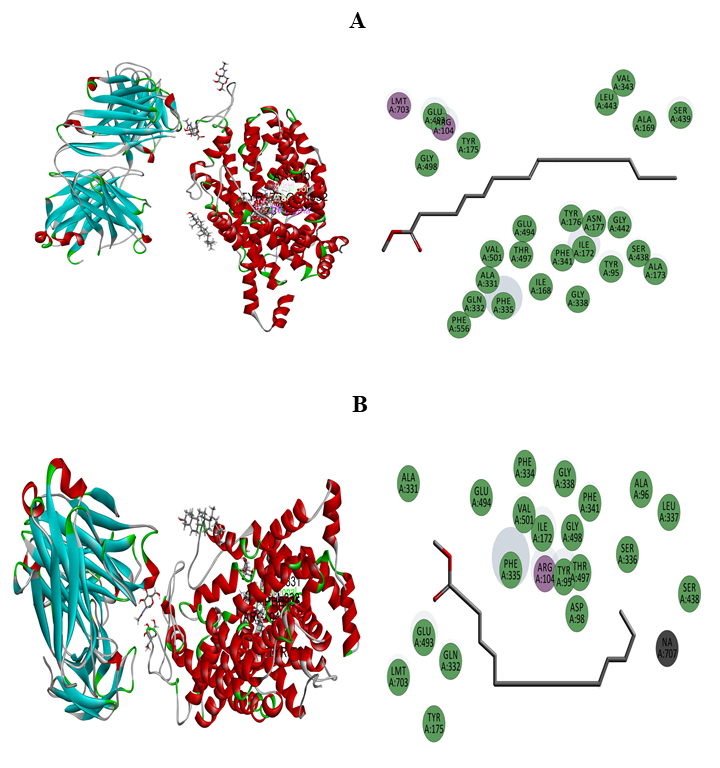
**

**Figure S13.** Best ranked poses and 2D interactions of **(A)** 9-Octadecenoic acid, methyl ester, (E)- **(B)** Hexadecanoic acid, methyl ester with human serotonin receptor (PDB: 5I6X) for antidepressant activity.
